# Supplementary material for: Is there a relationship between forest fires and deforestation in the Brazilian Amazon?
Source: PLoS One. 2024 Jun 28;19(6):e0306238. doi: 10.1371/journal.pone.0306238 (PMC11213292; doi:10.1371/journal.pone.0306238)
Supplement: Attachment — Submitted filename: Response to Reviewers 3.docx [file pone.0306238.s002.docx]

**Author's Response to Reviewers Comments 3**

Dear Editor and Reviewer.

We appreciate the speed in the manuscript evaluation process and the positive response to the scientific merit of the work. I hope that after these new considerations, the work fits within the highlighted criteria.

**Point 1:** We note that you state the following regarding your map data: "We appreciate the registrations made and the concern with intellectual property regarding Figures 1 and 4. It is important to highlight our dedication to following all legal, ethical, and compliance guidelines expressed in licenses, such as CC BY 4.0. We want to make it clear that these figures are the intellectual property of the authors, that is, we created them using the QGIS 3.2 software. Therefore, we did not remove the figures from the MAPBIOMAS website.

Spatialization and vegetation cover data are made available free of charge to the public by MAPBIOMAS, promoting transparency and allowing free access to crucial information for public policies, environmental monitoring, and territorial planning. What we did was interpolate this free data using the Kernel density estimation method, available in the QGIS 3.2 data processing tool (described in the methodology).

Therefore, the aforementioned institution will not sign this agreement, as it is not responsible for creating the images in this work, that is, the aforementioned images were not taken from MAPBIOMAS, being the exclusive intellectual property of the authors of this work."

However, the data from MAPBIOMAS is still copyrighted under CC BY-SA and as you used the data to create your maps, permission is still needed from the copyright holder to publish the map images using the copyrighted data.

**Response 1:** We are grateful for the records made and the care shown with the intellectual property of Figures 1 and 4. We would like to highlight that these figures are the intellectual property of the authors and that the uses of the aforementioned public data, as well as documents already forwarded, are by common consent in the national territory of Brazil.

However, to meet the requirements of this prestigious journal, we decided to remove the respective figures from the manuscript.

It is important to emphasize that this modification will not compromise the quality or the identification of the correlation between forest fires and deforestation, since the statistical and spatial conclusions remain unchanged.

Kind regards.
